# Measuring Large-Scale Social Networks with High Resolution

**DOI:** 10.1371/journal.pone.0095978

**Published:** 2014-04-25

**Authors:** Arkadiusz Stopczynski, Vedran Sekara, Piotr Sapiezynski, Andrea Cuttone, Mette My Madsen, Jakob Eg Larsen, Sune Lehmann

**Affiliations:** 1 DTU Compute, Technical University of Denmark, Kgs. Lyngby, Denmark; 2 The Niels Bohr Institute, University of Copenhagen, Copenhagen, Denmark; 3 Department of Anthropology, University of Copenhagen, Copenhagen, Denmark; University of Zaragoza, Spain

## Abstract

This paper describes the deployment of a large-scale study designed to measure human interactions across a variety of communication channels, with high temporal resolution and spanning multiple years—the Copenhagen Networks Study. Specifically, we collect data on face-to-face interactions, telecommunication, social networks, location, and background information (personality, demographics, health, politics) for a densely connected population of 1 000 individuals, using state-of-the-art smartphones as social sensors. Here we provide an overview of the related work and describe the motivation and research agenda driving the study. Additionally, the paper details the data-types measured, and the technical infrastructure in terms of both backend and phone software, as well as an outline of the deployment procedures. We document the participant privacy procedures and their underlying principles. The paper is concluded with early results from data analysis, illustrating the importance of multi-channel high-resolution approach to data collection.

## Introduction

Driven by the ubiquitous availability of data and inexpensive data storage capabilities, the concept of big data has permeated the public discourse and led to surprising insights across the sciences and humanities [1], [2]. While collecting data may be relatively easy, it is a challenge to combine datasets from multiple sources. This is in part due to mundane practical issues, such as matching up noisy and incomplete data, and in part due to complex legal and moral issues connected to data ownership and privacy, since many datasets contain sensitive data regarding individuals [3]. As a consequence, most large datasets are currently locked in ‘silos’, owned by governments or private companies, and in this sense the big data we use today are ‘shallow’—only a single or very few channels are typically examined.

Such shallow data limit the results we can hope to generate from analyzing these large datasets. We argue below (in Motivations Section) that in terms of understanding of human social networks, such shallow big data sets are not sufficient to push the boundaries in certain areas. The reason is that human social interactions take place across various communication channels; we seamlessly and routinely connect to the same individuals using face-to-face communication, phone calls, text messages, social networks (such as Facebook and Twitter), emails, and many other platforms. Our hypothesis is that, in order to understand social networks, we must study communication across these many channels that are currently siloed. Existing big data approaches have typically concentrated on large populations (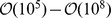), but with a relatively low number of bits per participant, for example in call detail records (CDR) studies [4] or Twitter analysis [5]. Here, we are interested in capturing deeper data, looking at multiple channels from sizable populations. Using big data collection and analysis techniques that can scale in number of participants, we show how to start deep, i.e. with detailed information about every single study participant, and then scale up to very large populations.

We are not only interested in collecting deep data from a large, highly connected population, but we also aim to create a dataset that is collected interactively, allowing us to change the collection process. This enables us to rapidly adapt and change our collection methods if current data, for example, have insufficient temporal resolution with regard to a specific question we would like to answer. We have designed our data collection setup in such a way that we are able to deploy experiments. We have done this because we know that causal inference is notoriously complicated in network settings [6]. Moreover, our design allows us to perform continuous quality control of the data collected. The mindset of real-time data access can be extended beyond pure research, monitoring data quality and performing interventions. Using the methods described here, we can potentially use big data in real time to observe and react to the processes taking place across entire societies. In order to achieve this goal, researchers must approach the data in the same way large Internet services do—as a resource that can be manipulated and made available in real time as this kind of data inevitably loses value over time.

In order to realize the interactive data collection, we need to build long-lasting testbeds to rapidly deploy experiments, while still retaining access to all the data collected hitherto. Human beings are not static; our behavior, our networks, our thinking change over time [7], [8]. To be able to analyze and understand changes over long time scales, we need longitudinal data, available not just to a single group of researchers, but to changing teams of researchers who work with an evolving set of ideas, hypotheses, and perspectives. Ultimately, we aim to be able to access the data containing the entire life-experience of people and look at their lives as dynamic processes. Eventually, we aim to even go beyond the lifespan of individuals and analyze the data of the entire generations. We are not there yet, but we are moving in this direction. For example, today, all tweets are archived in the Library of Congress (https://blog.twitter.com/2010/tweet-preservation), a person born today in a developed country has a good chance of keeping every single picture they ever take, the next generation will have a good chance of keeping highly detailed life-log, including, for example, every single electronic message they have ever exchanged with their friends. The status quo is that we need to actively opt out if we want to prevent our experiences from being auto-shared: major cloud storage providers offer auto-upload feature for pictures taken with a smartphone, every song we listen to on Spotify is remembered and used to build our profile—unless we actively turn on private mode.

In this paper, we describe a large-scale study that observes the lives of students through multiple channels—the Copenhagen Network Study. With its iterative approach to deployments, this study provides an example of an interdisciplinary approach. We collect data from multiple sources, including questionnaires, online social networks, and smartphones handed out to the students. Data from all of these channels are used to create a multi-layered view of the individuals, their networks, and their environments. These views can then be examined separately, and jointly, by researchers from different fields. We are building the Copenhagen Networks Study as a framework for long-lived extensible studies. The 2012 and 2013 deployments described here are called *SensibleDTU* and are based at the Technical University of Denmark. They have been designed as part of the *Social Fabric* project (see Acknowledgements for details) in close collaboration with researchers from the social sciences, natural sciences, medicine (public health), and the humanities. We are currently in the second iteration where we have deployed phones to about 1 000 participants, enabling us to compile a dataset of unprecedented size and resolution. In addition to the core task of collecting deep behavioral data, we also experiment with creating rich services for our participants and improving privacy practices.

Human lives, especially when seen over a period of months and years, take place in multiple dimensions. Capturing only a single channel, even for the entire life of an individual, limits the knowledge that can be applied to understand a human being. True interdisciplinary studies require deep data. Anthropologists, economists, philosophers, physicists, psychologists, public health researchers, sociologists, and computational social science researchers are all interested in distinct questions, and traditionally use very different methods. We believe that it is when these groups start working together, qualitatively better findings can be made.

Here we give a brief overview of the related work, in the domains of data collection and analysis, extend the description of the motivation driving the project, and outline the experimental plan and data collection methodology. We report on privacy and informed consent practices that are used in the study, emphasizing how we went beyond the usual practice in such studies and created some cutting edge solutions in the domain. We also report a few initial results from the project, primarily in the form of an overview of collected data, and outline future directions. We hope the work presented here will serve as a guideline for deploying similar massive sensor-driven human-data collection studies. With the overview of the collected data, we extend an invitation to researches of all fields to contact the authors for the purpose of defining novel projects around the Copenhagen Networks Study testbed.

## Related Work

Lazer et al. introduced computational social science (CSS) as a new field of research that studies individuals and groups in order to understand populations, organizations, and societies using big data, i.e. phone call records, GPS traces, credit card transactions, webpage visits, emails, and data from social networks [9]. CSS focuses on questions that can now be studied using data-driven computational analyses of datasets such as the ones mentioned above, and which could only previously be addressed as self-reported data or direct observations, for example dynamics in work groups, face-to-face interactions, human mobility, or information spreading. The hope is that such a data-driven approach will bring new types of insight that are not available using traditional methods. The challenges that emerge in this set of new approaches include wrangling big data, applying network analysis to dynamic networks, ensuring privacy of personal information, and enabling interdisciplinary work between computer science and social science, to name just a few.

In this section we describe related work in terms of the central methods of data collection. Furthermore, we provide a brief overview of results obtained from the analysis of CSS data, and finally, mention some principles regarding privacy and data treatment.

### Data collection

Many of the CSS studies carried out to date have been performed on call detail records (CDRs), which are records of phone calls and messages collected by mobile phone operators. Although CDRs can be a proxy for mobility and social interaction [10], much of the social interaction happens face-to-face, and may therefore be difficult to capture with CDRs or other channels such as social networks (Twitter, Facebook, etc.) [11]. To gain a fuller view of participants' behavior, some CSS studies have developed an approach of employing Radio Frequency Identification (RFID) devices [12], sociometetric badges [13], [14], as well as smartphones for the data collection [15]–[18]. Smartphones are unobtrusive, relatively cheap, feature a plethora of embedded sensors, and tend to travel nearly everywhere with their users. They allow for automatic collection of sensor data including GPS, WiFi, Bluetooth, calls, SMS, battery, and application usage [19]. However, collecting data with smartphones presents several limitations as sensing is mainly limited to pre-installed sensors, which may not be of highest quality. Furthermore, off-the-shelf software and hardware may not be sufficiently robust for longitudinal studies.

A large number of solutions for sensor-driven human data collection have been developed, ranging from dedicated software to complete platforms, notably ContextPhone [20], SocioXensor [21], MyExperience [22], Anonysense [23], CenceMe [24], Cityware [25], Darwin phones [26], Vita [27], and ContextToolbox [28].

Running longitudinal rich behavioral data collection from large populations presents multiple logistical challenges and only few studies have attempted to do this so far. In the Reality Mining study, data from 100 mobile phones were collected over a nine-month period [29]. In the Social fMRI study, 130 participants carried smartphones running the Funf mobile software [30] for 15 months [31]. Data was also collected from Facebook, credit card transactions, and surveys were pushed to the participants' phones. The Lausanne Data Collection Campaign [32], [33] featured 170 volunteers in the Lausanne area of Switzerland, between October 2009 and March 2011. In the SensibleOrganization study [34], researchers used RFID tags for a period of one month to collect face-to-face interactions of 22 employees working in a real organization. Preliminary results from the OtaSizzle study covering 20 participants from a large university campus have been reported [35]. Finally, in the Locaccino study [36], location within a metropolitan region was recorded for 489 participants for varying periods, ranging from seven days to several months.

### Data analysis

In the following, we provide selected examples of results obtained from analysis of CSS datasets in various domains.

#### Human Mobility

Gonzales et al. analyzed six months of CDRs of 100 000 users. Their results revealed that human mobility is quite predictable, with high spatial and temporal regularity, and few highly frequented locations [37]. Their findings were further explored by Song et al., who analyzed three months of CDRs from 50 000 individuals and found a 93% upper bound of predictability of human mobility. This figure applies to most users regardless of different travel patterns and demographics [38]. Sevtsuk et al. focused instead on the aggregate usage of 398 cell towers, describing the hourly, daily, and weekly patterns and their relation to demographics and city structure [39]. Bagrow et al. analyzed 34 weeks of CDRs for 90 000 users, identifying habitats (groups of related places) and found that the majority of individuals in their dataset had between 5 and 20 habitats [40]. De Domenico et al. showed in [41] how location prediction can be performed using multivariate non-linear time series prediction, and how accuracy can be improved considering the geo-spatial movement of other users with correlated mobility patterns.

#### Social Interactions

Face-to-face interactions can be used to model social ties over time and organizational rhythms in response to events [29], [42], [43]. Comparing these interactions with Facebook networks, Cranshaw et al. found that meetings in locations of high entropy (featuring a diverse set of visitors) are less indicative than meetings in locations visited by a small set of users [36]. Clauset et al. found that a natural time scale of face-to-face social networks is 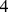 hours [44].

Onnela et al. analyzed CDRs from 3.9 million users [45] and found evidence supporting the weak ties hypothesis [46]. Lambiotte et al. analyzed CDRs from 2 million users and found that the probability of the existence of the links decreases as 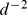, where 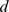 is the distance between users [47]. In another study with CDRs from 3.4 million users, the probability was found to decrease as 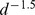 [48]. Analyzing CDRs for 2 million users, Hidalgo et al. found that persistent links tend to be reciprocal and associated with low degree nodes [49].

Miritello et al. analyzed CDRs for 20 million people and observed that individuals have a finite limit of number of active ties, and two different strategies for social communication [50], [51]. Sun et al. analyzed 20 million bus trips made by about 55% of the Singapore population and found distinct temporal patterns of regular encounters between strangers, resulting in a co-presence network across the entire metropolitan area [52].

#### Health and Public Safety

Using CDRs from the period of the 2008 earthquake in Rwanda, Kapoor et al. created a model for detection of the earthquake, the estimation of the epicenter, and determination of regions requiring relief efforts [53]. Aharony et al. performed and evaluated a fitness activity intervention with different reward schemes, based on face-to-face interactions [31], while Madan et al. studied how different illnesses (common cold, depression, anxiety) manifest themselves in common mobile-sensed features (WiFi, location, Bluetooth) and the effect of social exposure on obesity [54]. Salathé et al. showed that disease models simulated on top of proximity data obtained from a high school are in good agreement with the level of absenteeism during an influenza season [55], and emphasize that contact data is required to design effective immunization strategies.

#### Influence and Information Spread

Chronis et al. [16] and Madan et al. [56] investigated how face-to-face interactions affect political opinions. Wang et al. reported on the spread of viruses in mobile networks; Bluetooth viruses can have a very slow growth but can spread over time to a large portion of the network, while MMS viruses can have an explosive growth but their spread is limited to sub-networks [57]. Aharony et al. analyzed the usage of mobile apps in relation to face-to-face interactions and found that more face-to-face interaction increases the number of common applications [31]. Using RFID for sensing face-to-face interactions, Isella et al. estimated the most probable vehicles for infection propagation [58]. Using a similar technique, however applied to 232 children and 10 teachers in a primary school, Stehle et al. described a strong age homophily in the interactions between children [59].

Bagrow et al. showed how CDR communications, in relation to entertainment events (e.g. concerts, sporting events) and emergencies (e.g. fires, storms, earthquakes), have two well-distinguishable patterns in human movement [60]. Karsai et al. analyzed CDR from six millions users and found that strong ties tend to constrain the information spread within localized groups of individuals [61].

Studies of Christakis and Fowler on the spread of obesity and smoking in networks [62], [63] prompted a lively debate on how homophily and influence are confounded. Lyons was critical toward the statistical methods used [64]. Stelich et al. discussed how friendship formation in a dynamic network based on homophily can be mistaken for influence [65], and Shalizi and Thomas showed examples of how homophily and influence can be confounded [6]. Finally, Aral et al. provided a generalized statistical framework for distinguishing peer-to-peer influence from homophily in dynamic networks [66].

#### Socioeconomics and Organizational Behavior

For employees in a real work environment, face-to-face contact and email communication can be used to predict job satisfaction and group work quality [34]. Having more diverse social connections is correlated with economic opportunities, as found in the study containing CDRs of over 65 million users [67]. A similar result was reported in a study of economic status and physical proximity, where a direct correlation between more social interaction diversity and better financial status was found [31]. Or, as shown in a study of Belgian users, language regions in a country can be identified based solely on CDRs [68].

### Privacy

Data collected about human participants is sensitive and ensuring privacy of the participants is a fundamental requirement—even when participants may have limited understanding of the implications of data sharing [69], [70]. A significant amount of literature exists regarding the possible attacks that can be performed on personal data, such as unauthorized analysis [71] with a view to decoding daily routines [72] or friendships [42] of the participants. In *side channel information* attacks, data from public datasets (e.g. online social networks) are used to re-identify users [73]–[75]. Even connecting the different records of one user within the same system can compromise privacy [73]. Specific attacks are also possible in network data, as nodes can be identified based on the network structure and attributes of the neighbors [76], [77].

Various de-identification techniques can be applied to the data. *Personally Identifiable Information* (PII) is any information that can be used to identify an individual, such as name, address, social security number, date and place of birth, employment, education, or financial status. In order to avoid re-identification and consequent malicious usage of data, PII can be completely removed, hidden by aggregation, or transformed to be less identifiable, resulting in a trade-off between privacy and utility [78]. Substituting PII with the correspondent one-way hash allows removal of plaintext information and breaks the link to other datasets. This method, however, does not guarantee protection from re-identification [79]–[82]. 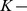
anonymity is a technique of ensuring that it is not possible to distinguish any user from at least 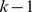 other in the dataset [83]; studies have shown that this method often may be too weak [72]. 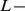
diversity [84] and 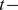
closeness [85] have been proposed as extensions of 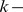
anonymity with stronger guarantees.

Another approach to introducing privacy is based on perturbing the data by introducing noise, with the goal of producing privacy-preserving statistics [86]–[90]. *Homomorphic encryption*, on the other hand, can be used to perform computation directly on the encrypted data, thus eliminating the need of exposing any sensitive information [91]–[94]; this technique has been applied, for example, to vehicle positioning data [95] and medical records [96].

The flows of data—creation, copying, sharing—can be restricted. *Information Flow Control* solutions such as [97]–[99] attempt to regulate the flow of information in digital systems. *Auditing* implementations such as [100]–[102] track the data flow by generating usage logs. *Data Expiration* makes data inaccessible after a specific time, for example by self-destruction or by invalidating encryption keys [103]–[106]. *Watermarking* identifies records using hidden fingerprints, to allow traceability and identification of leaks [107]–[109].

## Motivation

Here we describe our primary motivation for deploying the Copenhagen Networks Study, featuring deep and high-resolution data and a longitudinal approach.

### Multiplexity

The majority of big data studies use datasets containing data from a single source, such as call detail records (CDRs) [4], RFID sensors [110], Bluetooth scanners [111], or online social networks activity [2]. Although, as we presented in the Related Work section, analyzing these datasets has led to some exciting findings, we may however not understand how much bias is introduced in such single-channel approaches, particularly in the case of highly interconnected data such as social networks.

We recognize two primary concerns related to the single-source approach: incomplete data and limitation with respect to an interdisciplinary approach. For social networks, we intuitively understand that people communicate on multiple channels: they call each other on the phone, meet face-to-face, or correspond through email. Observing only one channel may introduce bias that is difficult to estimate [11]. Ranjan et al. investigated in [112] how CDR datasets, containing samples dependent upon user activity and requiring user participation, may bias our understanding of human mobility. The authors used data activities as the ground truth; due to applications running in the background, sending and requesting data, smartphones exchange data with the network much more often than typical users make calls and without the need for their participation. Comparing the number of locations and significant locations [113], they found that the CDRs reveal only a small fraction of users' mobility, when compared with data activity. The identified home and work locations, which are considered the most important locations, did not, however, differ significantly when estimated using either of the three channels (voice, SMS, and data).

Domains of science operate primarily on different types of data. Across the sciences, researchers are interested in distinct questions and use very different methods. Similarly, as datasets are obtained from different populations and in different situations, it is difficult to cross-validate or combine findings. Moreover, the single-channel origin of the data can be a preventive factor in applying expertise from multiple domains. If we collect data from multiple channels in the same studies, on the same population, we can work together across field boundaries and draw on the different expertise and results generated by the studies and thereby achieve more robust insights.

Social networks are ‘multiplex’ in the sense that many different types of links may connect any pair of nodes. While recent work [114], [115] has begun to explore the topic, a coherent theory describing multiplex, weighted, and directed networks remains beyond the frontier of our current understanding.

### Sampling

In many big data studies, data sampling is uneven. CDRs, for example, only provide data when users actively engage, by making or receiving a phone call or SMS. Users can also have different patterns of engagement with social networks, some checking and interacting several times a day, while others only do so once a week [116]. Further, CDRs are typically provided by a single provider who has a finite market share. If the market share is 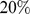 of the population and you consider only links internal to your dataset, this translates to only 4% of the total number of links, assuming random network and random sampling [4]. Thus, while CDRs might be sufficient when analysing of mobility, it is not clear that CDRs are a useful basis for social network analysis. Such uneven, sparse sampling decreases the resolution of data available for analysis. Ensuring the highest possible quality of the data, and even sampling, is possible with primarily passive data gathering, focusing on digital traces left by participants as they go through their lives, for example by using phones to automatically measure Bluetooth proximity, record location, and visible WiFi networks [9], [29], [31]. In cases where we cannot observe participants passively or when something simply goes wrong with the data collection, we aim to use the redundancy in the channels: if the participant turns off Bluetooth for a period, we can still estimate the proximity of participants using WiFi scans (as described in the Results section).

Uneven sampling not only reduces the quality of available data, but also—maybe more importantly—may lead to selection bias when choosing participants to include in the analysis. As investigated in [112], when only high-frequency voice-callers are chosen from a CDR dataset for the purpose of analysis, this can incur biases in Shannon entropy values (measure of uncertainty) of mobility, causing overestimation of the randomness of participants' behavior. Similarly, as shown in [116], choosing users with a large network and many interactions on Facebook may lead to overestimation of diversity in the ego-networks. Every time we have to discard a significant number of participants, we risk introducing bias in the data. Highly uneven sampling that cannot be corrected with redundant data, compels the researcher to make mostly arbitrary choices as part of the analysis, complicating subsequent analysis, especially when no well-established ground truth is available to understand the bias. Our goal here is to collect evenly sampled high-quality data for all the participants, so we do not have to discard anyone; an impossible goal, but one worth pursuing.

Since we only record data from a finite number of participants, our study population is also a subset, and every network we analyze will be sampled in some way, see [117] for a review on sampling. While the 2013 deployment produces a dataset that is nearly complete in terms of communication between the participants, it is clear that it is subject to other sampling-related issues. For example, a relatively small network embedded in a larger society has a large ‘surface’ of links pointing to the outside world, creating a *boundary specification problem* [118].

### Dynamics

The networks and behaviors we observe are not static; rather they display dynamics on multiple time-scales. Long-term dynamics may be lost in big data studies when the participants are not followed for a sufficiently long period, and only a relatively narrow slice of data is acquired. Short-term dynamics may be missed when the sampling frequency is too low.

It is a well-established fact that social networks evolve over time [8], [119]. The time scale of the changes varies and depends on many factors, for example the semester cycle in students' life, changing schools or work, or simply getting older. Without following such dynamics, and if we focus on a single temporal slice, we risk missing an important aspect of human nature. To capture it, we need long-term studies, that follow participants for months or even years.

**Figure 1:**
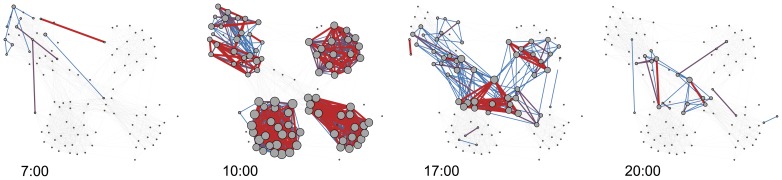
Dynamics of face-to-face interactions in the 2012 deployment. The participants meet in the morning, attend classes within four different study lines, and interact across majors in the evening. Edges are colored according to the frequency of observation, ranging from low (blue) to high (red). With 24 possible observations per hour, the color thresholds are respectively: blue (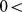 observations 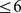), purple (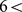 observations 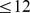), and red (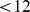 observations). Node size is linearly scaled according to degree.

Our behavior is not static, even when measured for very short intervals. We have daily routines, meeting with different people in the morning and hanging out with other people in the evening, see Figure 1. Our workdays may see us going to places and interacting with people differently than on weekends. It is easy to miss dynamics like these when the quality of the data is insufficient, either because it has not been sampled frequently enough or because of poor resolution, requiring large time bins.

Because each node has a limited bandwidth, only a small fraction of the network is actually ‘on’ at any given time, even if the underlying social network is very dense. Thus, to get from node A to node B, a piece of information may only travel on links that are active at subsequent times. Some progress has been made on the understanding of dynamic networks, for a recent review see [120]. However, in order to understand the dynamics of our highly dense, multiplex network, we need to expand and adapt the current methodologies, for example by adapting the link-based viewpoint to dynamical systems.

### Feedback

In many studies, the data collection phase is separated from the analysis. The data might have been collected during usual operation, before the idea of the study had even been conceived (e.g. CDRs, WiFi logs), or access to the data might have not been granted before a single frozen and de-identified dataset was produced.

One real strength of the research proposed here is that, in addition to the richness of the collected data, we are able to run controlled experiments, including surveys distributed via the smartphone software. We can, for example, divide participants into sub-populations and expose them to distinct stimuli, addressing the topic of causality as well as confounding factors both of which have proven problematic [64], [121] for the current state-of-the-art [122], [123].

Moreover, we monitor the data quality not only on the most basic level of a participant (number of data points) but also by looking at the entire live dataset to understand if the quality of the collected data is sufficient to answer our research questions. This allows us to see and fix bugs in the data collection software, or learn that certain behaviors of the participants may introduce bias in the data: for example after discovering missing data, some interviewed students reported turning their phones off for the night to preserve battery. This allowed us to understand that, even if in terms of the raw numbers, we may be missing some hours of data per day for these specific participants, there was very little information in that particular data anyway.

Building systems with real-time data processing and access allows us to provide the participants with applications and services. It is an important part of the study not only to collect and analyze the data but also to learn how to create a feedback loop, directly feeding back extracted knowledge on behavior and interactions to the participants. We are interested in studying how personal data can be used to provide feedback about individual behavior and promote self-awareness and positive behavior change, which is an active area of research in Personal Informatics [124]. Applications for participants create value, which may be sufficient to allow us to deploy studies without buying a large number of smartphones to provide to participants. Our initial approach has included the development and deployment of a mobile app that provides feedback about personal mobility and social interactions based on personal participant data [125]. Preliminary results from the deployment of the app, participant surveys, and usage logs suggest an interest in such applications, with a subset of participants repeatedly using the mobile app for personal feedback [126]. It is clear that feedback can potentially influence the study results: awareness of a certain behavior may cause participants to want to change that behavior. We believe, however, that such feedback is unavoidable in any study, and studying the effects of such feedback (in order to account for it) is an active part of our research.

### New Science

The ability to record the highly dynamic networks opens up a new, microscopic level of observation for the study of diffusion on the network. We are now able to study diffusion of behavior, such as expressions of happiness, academic performance, alcohol and other substance abuse, information, as well as real world infectious disease (e.g. influenza). Some of these vectors may spread on some types of links, but not others. For example, influenza depends on physical proximity for its spread, while information may diffuse on all types of links; with the deep data approach we can study differences and similarities between various types of spreading and the interplay between the various communication channels [127], [128].

A crucial step when studying the structure and dynamics of networks is to identify communities (densely connected groups of nodes) [129], [130]. In social networks, communities roughly correspond to social spheres. Recently, we pointed out that communities in many real world networks display *pervasive overlap*, where each and every node belongs to more than one group [131]. It is important to underscore that the question of whether or not communities in networks exhibit pervasive overlap has great practical importance. For example, the patterns of epidemic spreading change, and the optimal corresponding societal countermeasures are very different, depending on the details of the network structure.

Although algorithms that detect disjoint communities have operated successfully since the notion of graph partitioning was introduced in the 1970s [132], we point out that most networks investigated so far are highly incomplete in multiple senses. Moreover, we can use a simple model to show that sampling could cause pervasively overlapping communities to appear to be disjoint [133]. The results reveal a fundamental problem related to working with incomplete data: *Without an accurate model of the structural ordering of the full network, we cannot estimate the implications of working with incomplete data*. Needless to say, this fact is of particular importance to studies carried out on (thin) slices of data, describing only a single communication channel, or a fraction of nodes using that channel. By creating a high-quality, high-resolution data set, we are able to form accurate descriptions of the full data set needed to inform a proper theory for incomplete data. A deeper understanding of sampling is instrumental for unleashing the full potential of data from the billions of mobile phones in use today.

## Methods: Data Collection

The Copenhagen Networks Study aims to address the problem of single-modality data by collecting information from a number of sources that can be used to build networks, study social phenomena, and provide context necessary to interpret the findings. A series of questionnaires provides information on the socioeconomic background, psychological traces, and well-being of the participants; Facebook data enables us to learn about the presence and activity of subjects in the biggest online social networking platform [134]; finally, the smartphones carried by all participants record their location, telecommunication patterns, and face-to-face interactions. Sensor data is collected with fixed intervals, regardless of the users' activity, and thus the uneven sampling issue, daunting especially CDR-based studies, is mainly overcome. Finally, the study is performed on the largest and the most dense population to date in this type of studies. The physical density of the participants helps to address the problem of missing data, but raises new questions regarding privacy, since missing data about a person can, in many cases, be inferred from existing data of other participants. For example, if we know that person 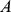, 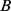, and 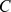 met at a certain location based on the data from person 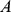, we do not need social and location data from 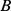 and 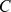 to know where and with whom they were spending time.

Below we describe the technical challenges and solutions in multi-channel data collection in 2012 and 2013 deployments. Data collection, anonymization, and storage were approved by the Danish Data Protection Agency, and comply with both local and EU regulations.

### Data Sources

The data collected in the two studies were obtained from questionnaires, Facebook, mobile sensing, an anthropological field study, and the WiFi system on campus.

#### Questionnaires

In 2012 we deployed a survey containing 95 questions, covering socioeconomic factors, participants' working habits, and the Big Five Inventory (BFI) measuring personality traits [135]. The questions were presented as a Google Form and participation in the survey was optional.

In 2013 we posed 310 questions to each participant. These questions were prepared by a group of collaborating public health researchers, psychologists, anthropologists, and economists from the Social Fabric project (see Acknowledgements). The questions in the 2013 deployment included BFI, Rosenberg Self Esteem Scale [136], Narcissism NAR-Q [137], Satisfaction With Life Scale [138], Rotters Locus of Control Scale [139], UCLA Loneliness scale [140], Self-efficacy [141], Cohens perceived stress scale [142], Major Depression Inventory [143], The Copenhagen Social Relation Questionnaire [144], and Panas [145], as well as number of general health- and behavior-related questions. The questions were presented using a custom-built web application, which allowed for full customization and complete control over privacy and handling of the respondents' data. The questionnaire application is capable of presenting different types of questions, with branching depending on the answers given by the participant, and saving each participant's progress. The application is available as an open source project at github.com/MIT-Model-Open-Data-and-Identity-System/SensibleDTUData-Apps-Questionaires. Participation in the survey was required for taking part in the experiment. In order to track and analyze temporal development, the survey (in a slightly modified form) was repeated every semester on all participating students.

#### Facebook Data

For all participants in both the 2012 and 2013 deployment, it was optional to authorize data collection from Facebook, and a large majority opted in. In the 2012 deployment, only the friendship graph was collected every 24 hours, until the original tokens expired. In the 2013 deployment, data from Facebook was collected as a snapshot, every 24 hours. The accessed scopes were birthday, education, feed, friend lists, friend requests, friends, groups, hometown, interests, likes, location, political views, religion, statuses, and work. We used long-lived Facebook access tokens, valid for 60 days, and when the tokens expired, participants received notification on their phones, prompting them to renew the authorizations. For the academic study purposes, the Facebook data provided rich demographics describing the participants, their structural (friendship graph) and functional (interactions) networks, as well as location updates.

#### Sensor Data

For the data collection from mobile phones, we used a modified version of the Funf framework [31] in both deployments. The data collection app was built using the framework runs on Android smartphones, which were handed out to participants (Samsung Galaxy Nexus in 2012 and LG Nexus 4 in 2013). All the bugfixes and the improvement of the framework are public and available under the OpenSensing github organization at github.com/organizations/OpenSensing.

In the 2012 deployment, we manually kept track of which phone was used by each student, and identified data using device IMEI numbers, but this created problems when the phones were returned and then handed out to other participants. Thus, in the 2013 deployment, the phones were registered in the system by the students in an OAuth2 authorization flow initiated from the phone; the data were identified by a token stored on the phone and embedded in the data files. The sensed data were saved as locally encrypted sqlite3 databases and then uploaded to the server every 2 hours, provided the phone was connected to WiFi. Each file contained 1 hour of participant data from all probes, saved as a single table. When uploaded, the data was decrypted, extracted, and included in the main study database.

#### Qualitative Data

An anthropological field study was included in the 2013 deployment. An anthropologist from the Social Fabric project was embedded within a randomly selected group of approximately 60 students (August 2013–august 2014). A field study consists of participant observation within the selected group, collecting qualitative data while simultaneously engaging in the group activities. The goal is to collect data on various rationales underlying different group formations, while at the same time experiencing bodily and emotionally what it was like to be part of these formations [146]. The participant observation included all the student activities and courses, including extracurricular activities such as group work, parties, trips, and other social leisure activities. All participants were informed and periodically reminded about the role of the anthropologist.

In addition to its central purpose, the anthropological data adds to the multitude of different data channels, deepening the total pool of data. This proved useful for running and optimizing the project in a number of ways.

Firstly, data from qualitative social analysis are useful—in a very practical sense—in terms of acquiring feedback from the participants. One of the goals of the project is to provide value to the participants; in addition to providing quantified-self style access to data, we have also created a number of public services: a homepage, a Facebook page, and a blog, where news and information about the project can be posted and commented on. These services are intended to keep the students interested, as well as to make participants aware of the types and amounts of data collected (see Privacy section). Because of the anthropologist's real-world engagement with the students, the qualitative feedback contains complex information about participants' interests and opinions, including what annoyed, humored, or bored them. This input has been used to improve existing services, such as visualizations (content and visual expression), and to develop ideas for the future services. In summary, qualitative insights helped us understand the participants better and, in turn, to maintain and increase participation.

Secondly, the inclusion of qualitative data increases the potential for interdisciplinary work between the fields of computer science and social science. Our central goal is to capture the full richness of social interactions by increasing the number of recorded communication channels. Adding a qualitative social network approach makes it possible to relate the qualitative observations to the quantitative data obtained from the mobile sensing, creating an interdisciplinary space for methods and theory. We are particularly interested in the relationship between the observations made by the embedded anthropologist and the data recorded using questionnaires and mobile sensing, to answer questions about the elements difficult to capture using our high-resolution approach. Similarly, from the perspective of social sciences, we are able to consider what may be captured by incorporating quantitative data from mobile sensing into a qualitative data pool—and what can we learn about social networks using modern sensing technology.

Finally, these qualitative data can be used to ground the mathematical modeling process. Certain things are difficult or impossible to infer from quantitative measurements and mathematical models of social networks, particularly in regard to understanding *why* things happen in the network, as computational models tend to focus on *how*. Questions about relationship-links severing, tight networks dissolving, and who or what caused the break, can be very difficult to answer, but they are important with regard to understanding the dynamics of the social network. By including data concerned with answering *why* in social networks, we add a new level of understanding to the quantitative data.

#### WiFi Data

For the 2012 deployment, between August 2012 and May 2013, we were granted access to the campus WiFi system logs. Every 10 minutes the system provided metadata about all devices connected to the wireless access points on campus (access point MAC address and building location), together with the student ID used for authentication. We collected the data in a de-identified form, removing the student IDs and matching the participants with students in our study. Campus WiFi data was not collected for the 2013 deployment.

### Backend System

The backend system, used for data collection, storage, and access, was developed separately for the 2012 and 2013 deployments. The system developed in 2012 was not designed for extensibility, as it focused mostly on testing various solutions and approaches to massive sensor-driven data collection. Building on this experience, the system for the 2013 deployment was designed and implemented as an extensible framework for data collection, sharing, and analysis.

#### The 2012 Deployment

The system for the 2012 deployment was built as a Django web application. The data from the participants from the multiple sources, were stored in a CouchDB database. The informed consent was obtained by presenting a document to the participants after they authenticated with university credentials. The mobile sensing data was stored in multiple databases inside a single CouchDB instance and made available via an API. Participants could access their own data, using their university credentials. Although sufficient for the data collection and research access, the system performance was not adequate for exposing the data for real-time application access, mainly due to the inefficient de-identification scheme and insufficient database structure optimization.

#### The 2013 Deployment

**Figure 2:**
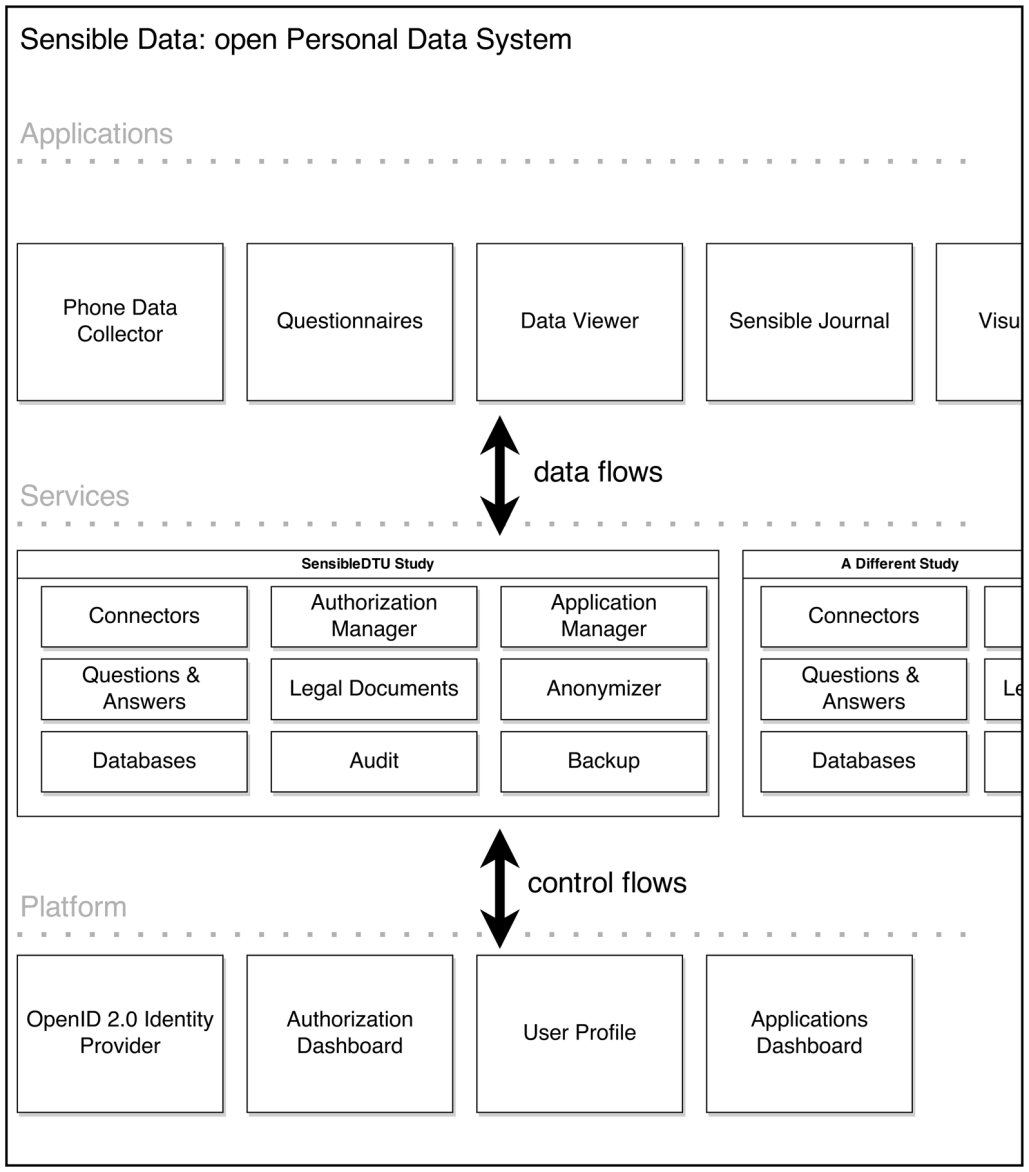
Sensible Data openPDS architecture. This system is used in the 2013 deployment and consists of three layers: platform, services, and applications. The platform contains element common for multiple services (in this context: studies). The studies are the deployments of particular data collection efforts. The applications are OAuth2 clients to studies and can submit and access data, based on user authorizations.

The 2013 system was built as an open Personal Data System (openPDS) [147] in an extensible fashion. The architecture of the system is depicted in Figure 2 and consisted of three layers: platform, services, and applications. In the platform layer, the components common for multiple services were grouped, involving identity provider and participant-facing portal for granting authorizations. The identity provider was based on OpenID 2.0 standard and enabled single sign-on (SSO) for multiple applications. The authorizations were realized using OAuth2 and could be used with both web and mobile applications. Participants enroll into studies by giving informed consent and subsequently authorizing application to submit and access data from the study. The data storage was implemented using MongoDB. Participants can see the status and change their authorizations on the portal site, the system included an implementation of the Living Informed Consent [3].

### Deployment Methods

Organizing studies of this size is a major undertaking. All parts from planning to execution have to be synchronized, and below we share some considerations and our approaches. While their main purpose was identical, the two deployments differed greatly in size and therefore also in the methods applied for enrolling and engaging the participants.

#### SensibleDTU 2012

In 2012 approximately 1,400 new students were admitted to the university, divided between two main branches of undergraduate programs. We focused our efforts on the larger branch containing 900 students, subdivided into 15 study lines (majors). For this deployment we had 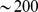 phones available to distribute between the students. To achieve maximal coverage and density of the social connections, we decided to only hand out phones in a few selected majors that had a sufficient number of students interested in participating in the experiment. Directly asking students about their interest in the study was not a good approach, as it could lead to biased estimates and would not scale well for a large number of individuals. Instead, we appealed to the competitive element of human nature by staging a competition, running for two weeks from the start of the semester. All students had access to a web forum, which was kept separate for each major, where they could post ideas that could be realized by the data we would collect, and subsequently vote for their own ideas or three seed ideas that we provided. The goal of the competition was twofold; first we wanted students to register with their Facebook account, thereby enabling us to study their online social network, and second we wanted to see which major could gain most support (percentage of active students) behind a single idea. Students were informed about the project and competition by the Dean in person and at one of 15 talks given—one at each major. Students were told that our choice of participants would be based on the support each major could muster behind their strongest idea before a given deadline. This resulted in 24 new research ideas and 1 026 unique votes. Four majors gained 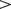
93% support for at least one idea and were chosen to participate in the experiment.

The physical handing out of the phones was split into four major sessions, in which students from the chosen majors were invited; additional small sessions were arranged for students that were unable to attend the main ones. At each session, participants were introduced to our data collection methods, de-identification schemes, and were presented with the informed consent form. In addition, the participants were instructed to fill out the questionnaire. A small symbolic deposit in cash was requested from each student; this served partially as compensation for broken phones, but was mainly intended to encourage participants take better care of the phones, than if they had received them for free [148]. Upon receiving a phone, participants were instructed to install the data collector application. The configuration on each phone was manually checked when participants were leaving—this was particularly important to ensure high quality of data.

This approach had certain drawbacks; coding and setting up the web fora, manually visiting all majors and introducing them to the project and competition, and organizing the handout sessions required considerable effort and time. However, certain aspects were facilitated with strong support from the central administration of the university. A strong disadvantage of the outlined handout process is that phones were handed out 3–4 weeks into the semester, thus missing the very first interactions between students.

#### SensibleDTU 2013

The 2013 deployment was one order of magnitude larger, with 1 000 phones to distribute. Furthermore, our focus shifted to engaging the students as early as possible. Pamphlets informing prospective undergraduate students about the project were sent out along with the official acceptance letters from the university. Early-birds who registered online via Facebook using the links given in the pamphlet were promised phones before the start of their studies. Students from both branches of undergraduate programs were invited to participate (approximately 1 500 individuals in total), as we expected an adoption percentage between 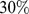 and 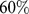. Around 300 phones were handed out to early-birds, and an additional 200 were handed out during the first weeks of semester. As the adoption rate plateaued, we invited undergraduate students from older years to participate in the project.

The structure of the physical handout was also modified, the participants were requested to enroll online before receiving the phone. Moreover, the informed consent and the questionnaire were part of the registration. Again, we required a symbolic cash deposit for each phone. We pre-installed custom software on each phone to streamline the handout process; students still had to finalize set up of the phones (make them Bluetooth-discoverable, activate WiFi connection, etc.).

For researchers considering similar projects with large scale handouts, we recommend that the pool of subjects are engaged in the projects as early as possible and be sure to keep their interest. Make it easy for participants to contact you, preferably through media platforms aimed at their specific age group. Establish clear procedures in case of malfunctions. On a side note, if collecting even a small deposit, when multiplied by a factor of 1 000, the total can add up to significant amount, which must be handled properly.

## Methods: Privacy

When collecting data of very high resolution, over an extended period, from a large population, it is crucial to address the privacy of the participants appropriately. We measure the privacy as a difference between what a participant understands and consents to regarding her data, and what in fact happens to these data.

We believe that ensuring sufficient privacy for the participants, in large part, is the task of providing them with tools to align the data usage with their understanding. Such privacy tools must be of two kinds: to inform, ensuring participants understand the situation, and to control, aligning the situation with the participant's preferences. There is a tight loop where these tools interact: as the participant grows more informed, she may decide to change the settings, and then verify if the change had the expected result. By exercising the right to information and control, the participant expresses Living Informed Consent as described in [3].

Not all students are interested in privacy, in fact we experienced quite the opposite attitude. During our current deployments the questions regarding privacy were rarely asked by the participants, as they tended to accept any terms presented to them without thorough analysis. It is our—the researchers'—responsibility to make the participants more aware and empowered to make the right decisions regarding their privacy: by providing the tools, promoting their usage, and engaging in a dialog about privacy-related issues.

In the 2012 deployment, we used a basic informed consent procedure with an online form accepted by the participants, after they authenticated with the university account system. The accepted form was then stored in a database, together with the username, timestamp, and the full text displayed to the participant. The form itself was a text in Danish, describing the study purpose, parties responsible, and participants' rights and obligations. The full text is available at [149] with English translation available at [150].

In the 2013 deployment, we used our backend solution (described in Backend System Section) to address the informed consent procedure and privacy in general. The account system, realized as an OpenID 2.0 server, allowed us to enroll participants, while also supporting research and developer accounts (with different levels of data access). The sensitive Personally Identifiable Information attributes (PIIs) of the participants were kept completely separate from the participant data, all the applications identified participants based only on the pseudonym identifiers. The applications could also access a controlled set of identity attributes for the purpose of personalization (e.g. greeting the participant by name), subject to user OAuth2 authorization. In the enrollment into the study, after the participant had accepted the informed consent document—essentially identical to that from 2012 deployment—a token for a scope *enroll* was created and shared between the platform and service (see Figure 2). The acceptance of the document was recorded in the database by storing the username, timestamp, hash of the text presented to the participant, as well as the git commit identifying the version of the form.

**Figure 3:**
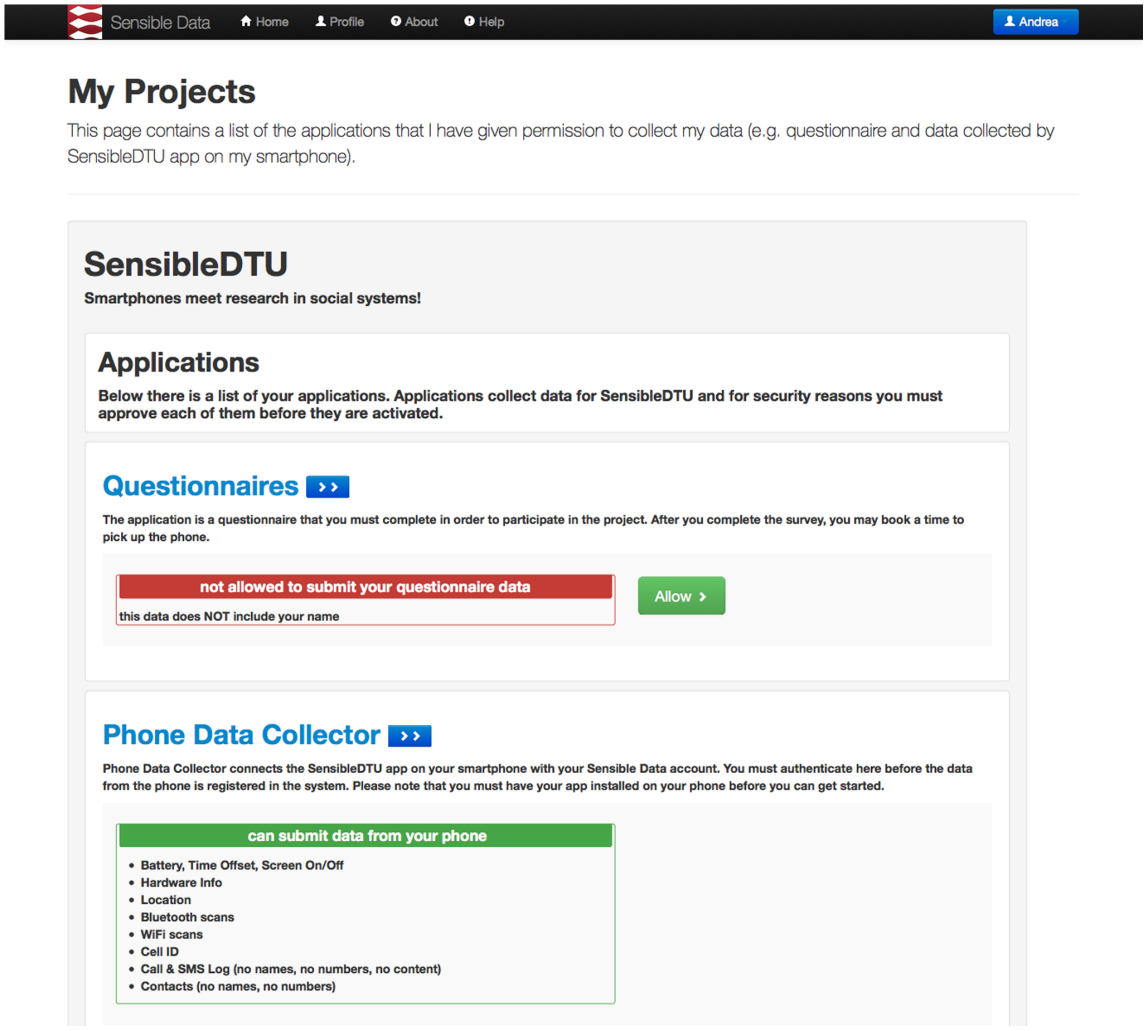
Authorizations page. Participants have an overview of the studies in which they are enrolled and which applications are able to submit to and access their data. This is an important step towards users' understanding what happens with their data and to exercising control over it. This figure shows a translated version of the original page that participants saw in Danish.

All the communication in the system was realized over HTTPS, and endpoints were protected with short-lived OAuth2 bearer tokens. The text of the documents, including informed consent, was stored in a git repository, allowing us to modify everything, while still maintaining the history and being able to reference which version each participant has seen and accepted. A single page overview of the status of the authorizations, presented in Figure 3, is an important step in moving beyond lengthy, incomprehensible legal documents accepted by the users blindly and giving more control over permissions to the participant.

**Figure 4:**
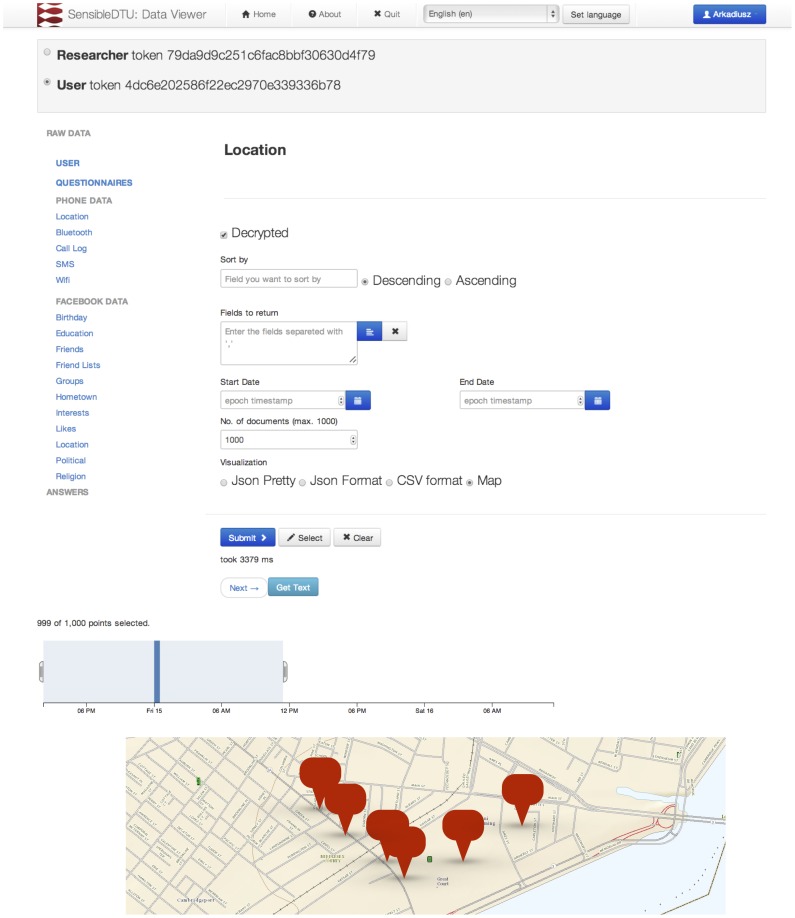
Data viewer application. All the collected data can be explored and accessed via an API. The API is the same for research, application, and end-user access, the endpoints are protected by OAuth2 bearer token. Map image from USGS National Map Viewer, replacing original image used in the deployed application (Google Maps).

In the 2013 deployment, the participants could access all their data using the same API as the one provided for the researchers and application developers. To simplify the navigation, we developed a data viewer application as depicted in Figure 4, which supports building queries with all the basic parameters in a more user-friendly way than constructing API URLs. Simply having access to all the raw data is, however, not sufficient, as it is really high-level inferences drawn from the data that are important to understand, for example *Is someone accessing my data to see how fast I drive or to study population mobility*? For this purpose, we promoted the development of a *question & answer* framework, where the high-level features are extracted from the data before leaving the server, promoting better participant understanding of data flows. This is aligned with the vision of the open Personal Data Store [147].

Finally, for the purposes of engaging the participants in the discussion about privacy, we published blogposts (e.g. https://www.sensible.dtu.dk/?p=1622), presented relevant material to students, and answered their questions via the Facebook page(https://www.facebook.com/SensibleDtu).

## Results and Discussion

As described in the previous sections, our study has collected comprehensive data about a number of aspects regarding human behavior. Below, we discuss primary data channels and report some early results and findings. The results are mainly based on the 2012 deployment due to the availability of longitudinal data.

### Bluetooth and Social Ties

Bluetooth is a wireless technology ubiquitous in modern-day mobile devices. It is used for short-range communication between devices, including smartphones, hands-free headsets, tablets, and other wearables. As the transmitters used in mobile devices are primarily of very short range—between 5 and 10 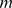 (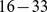 feet)—detection of the devices of other participants (set in ‘visible’ mode) can be used as a proxy for face-to-face interactions [29]. We take the individual Bluetooth scans in the form 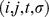, denoting that device 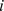 has observed device 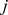 at time 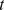 with signal strength 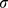. Bluetooth scans do not constitute a perfect proxy for face-to-face interactions [151], since a) it is possible for people within 10 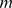 radius not to interact socially, and b) it is possible to interact socially over a distance greater than 10 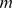, nevertheless, they have been successfully used for sensing social networks [31] or crowd tracking [152].

Between October 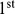, 2012 and September 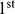, 2013, we collected 12 623 599 Bluetooth observations in which we observed 153 208 unique devices. The scans on the participants' phones were triggered every five minutes, measured from the last time the phone was powered on. Thus, the phones scanned for Bluetooth in a desynchronized fashion, and not according to a global schedule. To account for this, when extracting interactions from the raw Bluetooth scans, we bin them into fixed-length time windows, aggregating the scans within them. The resulting adjacency matrix, 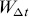 does not have to be strictly symmetric, meaning that participant 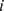 can observe participant 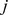 in time-bin 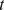, but not the other way around. Here we assume that Bluetooth scans do not produce false positives (devices are not discovered unless they are really there), and in the subsequent network analysis, we force the matrix to be symmetric, assuming that if participant 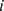 observed participant 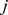, the opposite is also true.

**Figure 5:**
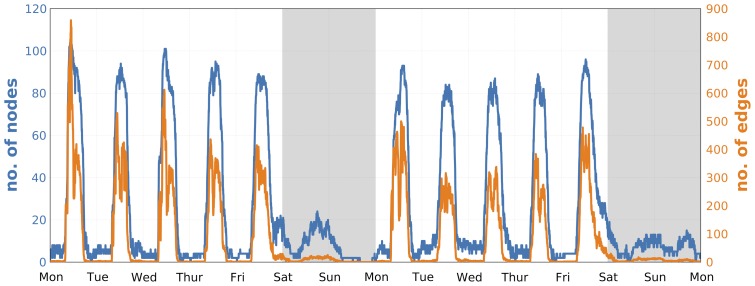
Weekly temporal dynamics of interactions. Face-to-face interaction patterns of participants in 5-minute time-bins over two weeks. Only active participants are included, i.e. those that have either observed another person or themselves been observed in a given time-bin. On average we observed 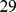 edges and 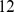 nodes in 5-minute time-bins and registered 10 634 unique links between participants.

The interactions between the participants exhibit both daily and weekly rhythms. Figure 1 shows that the topology of the network of face-to-face meetings changes significantly within single day, revealing academic and social patterns formed by the students. Similarly, the intensity of the interactions varies during the week, see Figure 5.

**Figure 6:**
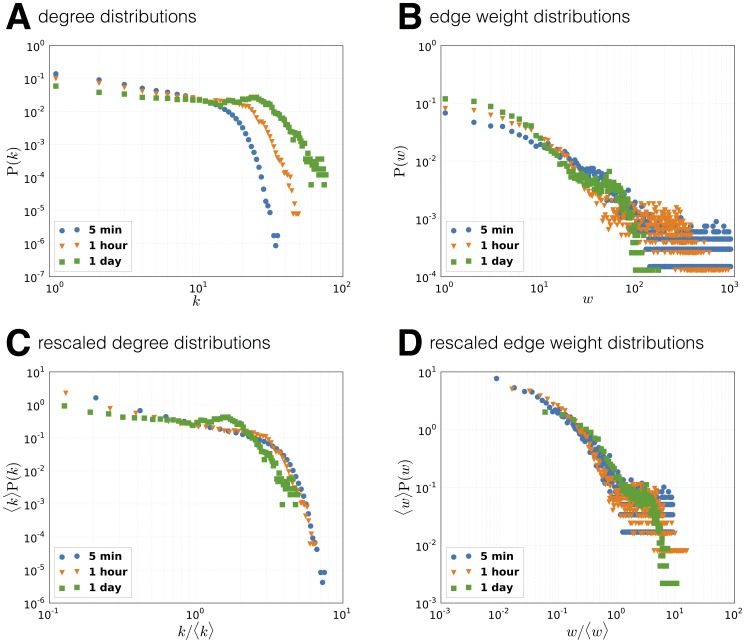
Face-to-face network properties at different resolution levels. Distributions are calculated by aggregating sub-distributions across temporal window. Differences in rescaled distributions suggest that social dynamics unfold on multiple timescales.

Aggregating over large time-windows blurs the social interactions (network is close to fully connected) while a narrow window reveals detailed temporal structures in the network. Figure 6A shows the aggregated degree distributions for varying temporal resolutions, with 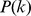 being shifted towards higher degrees for larger window sizes; this is an expected behavior pattern since each node has more time to amass connections. Figure 6B presents the opposite effect, where the edge weight distributions 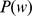 shift towards lower weights for larger windows; this is a consequence on definition of a link for longer time-scales or, conversely, of links appearing in each window on shorter timescales. To compare the distribution between timescales, we rescale the properties according to Krings et al. [153] as 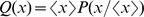 with 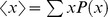 (Figure 6C and 6D). The divergence of the rescaled distributions suggest a difference in underlying social dynamics between long and short timescales, an observation supported by recent work on temporal networks [44], [153], [154].

### WiFi as an Additional Channel for Social Ties

Over the last two decades, wireless technology has transformed our society to the degree where every city in the developed world is now fully covered by mobile [155] and wireless networks [156]. The data collector application for mobile phones was configured to scan for wireless networks in constant intervals, but also to record the results of scans triggered by any other application running on the phone (‘opportunistic’ sensing). Out of the box, Android OS scans for WiFi every 15 seconds, and since we collected these data, our database contains 42 692 072 WiFi observations, with 142 871 unique networks (SSIDs) between October 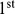, 2012 and September 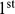, 2013 (i.e. the 2012 deployment). Below we present the preliminary result on WiFi as an additional data-stream for social ties, to provide an example of how our multiple layers of information can complement and enrich each other.

For computational social science, using Bluetooth-based detection of participants' devices as a proxy for face-to-face interactions is a well-established method [19], [29], [31]. The usage of WiFi as a social proxy has been investigated [157], but, to our knowledge, has not yet been used in a large-scale longitudinal study. For the method we describe here, the participants' devices do not sense each other, instead they record the visible beacons (in this instance WiFi access points) in their environment. Then, physical proximity between two devices—or lack thereof—can be inferred by comparing results of the WiFi scans that occurred within a sufficiently small time window. Proximity is assumed if the lists of access points (APs) visible to both devices are similar according to a similarity measure. We establish the appropriate definition of the similarity measure in a data-driven manner, based on best fit to Bluetooth data. The strategy is to compare the lists of results in 10-minute-long time bins, which corresponds to the forced sampling period of the WiFi probe as well as to our analysis of Bluetooth data. If there are multiple scans within the 10-minute bin, the results are compared pair-wise, and proximity is assumed if at least one of these comparisons is positive. The possibility of extracting face-to-face interactions from such signals is interesting, due to the ubiquitous nature of WiFi and high temporal resolution of the signal.

**Figure 7:**
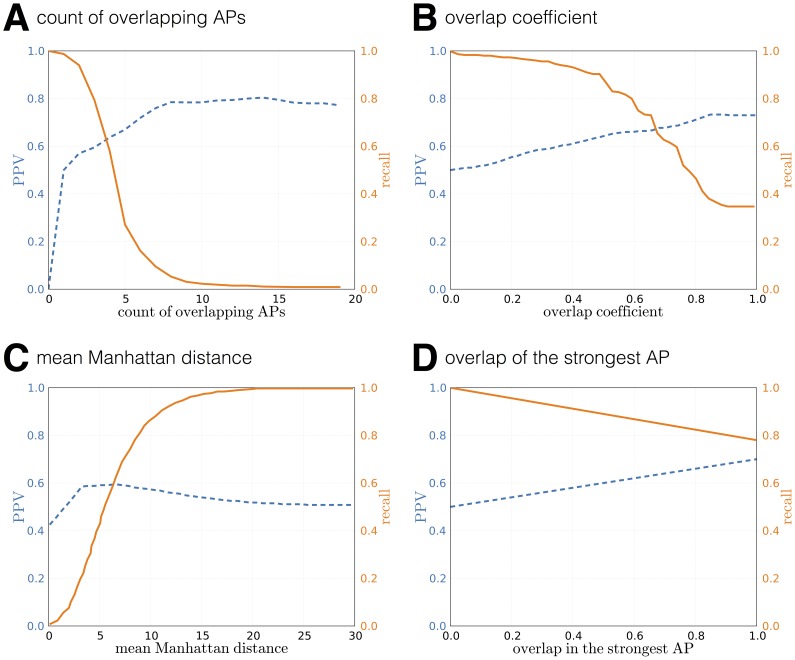
WiFi similarity measures. Positive predictive value (precision, ratio of number of true positives to number of positive calls, marked with dashed lines) and recall (sensitivity, fraction of retrieved positives, marked with solid lines) as functions of parameters in different similarity measures. **A**) In 98% of face-to-face meetings derived from Bluetooth, the two devices also sensed at least one common access point. **D**) Identical strongest access point for two separate mobile devices is a strong indication of a face-to-face meeting.

We consider four measures and present their performance in Figure 7. Figure 7A shows the positive predictive value and recall as a function of minimum number of overlapping access points (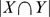) required to assume physical proximity. In approximately 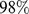 of all Bluetooth encounters, at least one access point was seen by both devices. However, the recall drops quickly with the increase of their required number. This measure favors interactions in places with a high number of access points, where it is more likely that devices will have a large scan overlap. The result confirms that lack of a common AP has a very high positive predictive power as a proxy for *lack* of physical proximity, as postulated in [158]. Note, that for the remaining measures, we assume at last one overlapping AP in the compared lists of scan results.

The overlap coefficient defined as 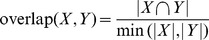 penalizes encounters taking place in WiFi-dense areas, due to higher probability of one device picking up a signal from a remote access point that is not available to the other device, see Figure 7B.

Next, we compare the received signal strengths between overlapping routers using the mean 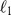-norm (mean Manhattan distance, 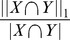). Received signal strength (RSSI) is measured in 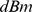 and the Manhattan distance between two routers is the difference in the RSSI between them, measured in 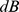. Thus, the mean Manhattan distance is the mean difference in received signal strength of the overlapping routers in the two compared scans.

Finally, we investigate the similarity based on the router with the highest received signal strength—the proximity is assumed whenever it is the same access point for both devices, 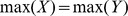. This measure provides both high recall and positive predictive value and, after further investigation for the causes for errors, is a candidate proxy for face-to-face interactions.

The performance of face-to-face event detection based on WiFi can be further improved by applying machine-learning approaches [158], [159]. It is yet to be established, by using longitudinal data, whether the errors in using single features are caused by inherent noise in measuring the environment, or if there is a bias that could be quantified and mitigated. Most importantly, the present analysis is a proof-of-concept and further investigation is required to verify if networks inferred from WiFi and Bluetooth signals are satisfyingly similar, before WiFi can be used as an autonomous channel for face-to-face event detection in the context of current and future studies. Being able to quantify the performance of multi-channel approximation of face-to-face interaction and to apply it in the data analysis is crucial to address the problem of missing data, as well as to estimate the feasibility and understand the limitations of single-channel studies.

### Location and Mobility

A number of applications ranging from urban planning, to traffic management, to containment of biological diseases rely on the ability to accurately predict human mobility. Mining location data allows extraction of semantic information such as points of interest, trajectories, and modes of transportation [160]. In this section we report the preliminary results of an exploratory data analysis of location and mobility patterns.

**Figure 8:**
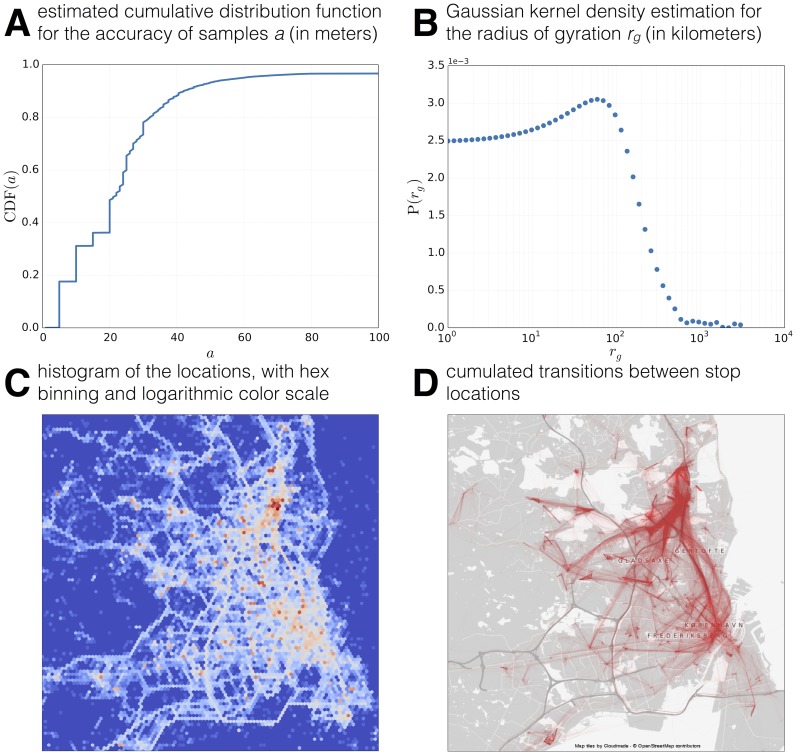
Location and Mobility. We show the accuracy of the collected samples, radius of gyration of the participants, and identify patterns of collective mobility.

Location data was obtained by periodically collecting the best position estimate from the location sensor on each phone, as well as recording location updates triggered by other applications running on the phone (opportunistic behavior). In total we collected 7 593 134 data points in 2012 deployment in the form (userid, timestamp, latitude, longitude, accuracy). The best-effort nature of the data presents new challenges when compared with the majority of location mining literature, which focuses on high-frequency, high-precision GPS data. Location samples on the smartphones can be generated by different providers, depending on the availability of the Android sensors, as explained in developer.android.com/guide/topics/location/strategies.html. For this reason, accuracy of the collected position can vary between a few meters for GPS locations, to hundreds of meters for cell tower location. Figure 8A shows the estimated cumulative distribution function for the accuracy of samples; almost 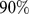 of the samples have a reported accuracy better than 40 meters.

We calculate the radius of gyration 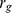 as defined in [38] and approximate the probability distribution function using a gaussian kernel density estimation, see Figure 8B. We select the appropriate kernel bandwidth through leave-one-out cross-validation scheme from Statsmodels KDEMultivariate class [161]. The kernel density peaks around 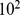 km and then rapidly goes down, displaying a fat-tailed distribution. Manual inspection of the few participants with 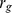 around 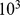 km revealed that travels abroad can amount to such high mobility. Although we acknowledge that this density estimation suffers due to the low number of samples, our measurements suggest that real participant mobility is underestimated in studies based solely on CDRs, such as in [38], as they fail to capture travels outside of the covered area.

Figure 8C shows a two-dimensional histogram of the locations, with hexagonal binning and logarithmic color scale (from blue to red). The red hotspots identify the most active places, such as the university campus and dormitories. The white spots are the frequently visited areas, such as major streets and roads, stations, train lines, and the city center.

From the raw location data we can extract stop locations as groups of locations clustered within distance 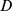 and time 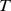 [162]–[165]. By drawing edges between stop locations for each participant, so that the most frequent transitions stand out, we can reveal patterns of collective mobility (Figure 8D).

### Call and Text Communication Patterns

With the advent of mobile phones in the late 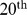 century, the way we communicate has changed dramatically. We are no longer restricted to landlines and are able to move around in physical space while communicating over long distances.

The ability to efficiently map communication networks and mobility patterns (using cell towers) for large populations has made it possible to quantify human mobility patterns, including investigations of social structure evolution [166], economic development [67], human mobility [37], [38], spreading patterns [57], and collective behavior with respect to emergencies [60]. In this study, we have collected call logs from each phone as (caller, callee, duration, timestamp, call type), where the call type could be incoming, outgoing, or missed. Text logs contained (sender, recipient, timestamp, incoming/outgoing, one-way hash of content).

In the 2012 deployment we collected 56 902 incoming and outgoing calls, of which 42 157 had a duration longer than zero seconds. The average duration of the calls was 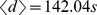, with a median duration of 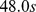. The average ratio between incoming and outgoing calls for a participant was 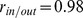. In the same period, we collected 161 591 text messages with the average ratio for a participant 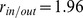.

**Figure 9:**
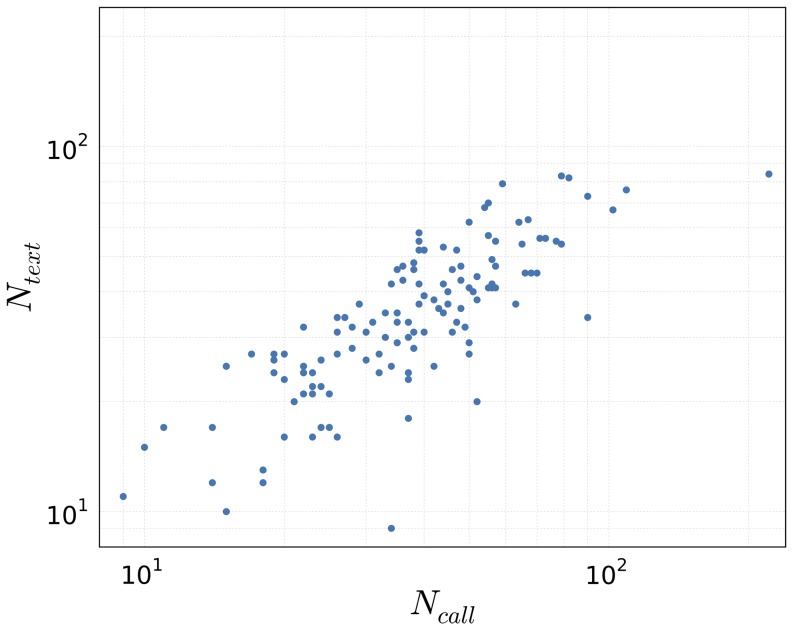
Diversity of communication logs. Diversity is estimated as the set of unique numbers that a person has contacted or been contacted by in the given time period on a given channel. We note a strong correlation in diversity (Pearson correlation of 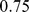, 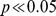), whereas the similarity of the sets of nodes is fairly low (on average 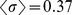).

We find a Pearson correlation of 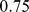 (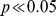) between the number of unique contacts participants contacted via SMS and voice calls, as depicted in Figure 9. However, the similarity 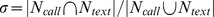 between the persons a participant contacts via calls (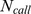) and SMS (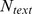) is on average 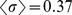, suggesting that even though participants utilize both forms of communication in similar capacity, those two are, in fact, used for distinct purposes.

**Figure 10:**
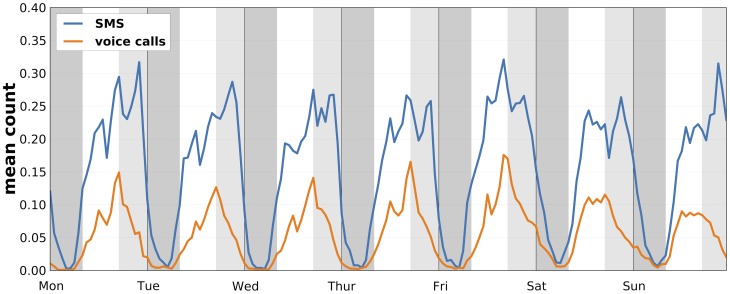
Weekly temporal dynamics of interactions. All calls and SMS, both incoming and outgoing, were calculated over the entire dataset and averaged per participant and per week, showing the mean number of interactions participants had in a given weekly bin. Light gray denotes 5pm, the time when lectures end at the university, dark gray covers night between 12 midnight and 8am. SMS is used more for communication outside regular business hours.

Figure 10 shows the communication for SMS and voice calls (both incoming and outgoing, between participants and with the external world) as a time series, calculated through the entire year and scaled to denote the mean count of interactions participants had in given hourly time-bins in the course of a week. Also here, we notice differences between the two channels. While both clearly show a decrease in activity during lunch time, call activity peaks around the end of the business day and drops until next morning. In contrast, after a similar decrease that we can associate with commute, SMS displays another evening peak. Also at night, SMS seems to be a more acceptable form of communication, with message exchanges continuing late and starting early, especially on Friday night, when the party never seems to stop.

We point out that the call and SMS dynamics display patterns that are quite distinct from face-to-face interactions between participants as seen in Figure 5. Although calls and SMS communication are different on the weekends, the difference is not as dramatic as in the face-to-face interactions between the participants. This indicates that the face-to-face interactions we observe during the week are driven primarily by university-related activities, and only few of these ties manifest themselves during the weekends, despite the fact that the participants are clearly socially active, sending and receiving calls and messages.

**Figure 11:**
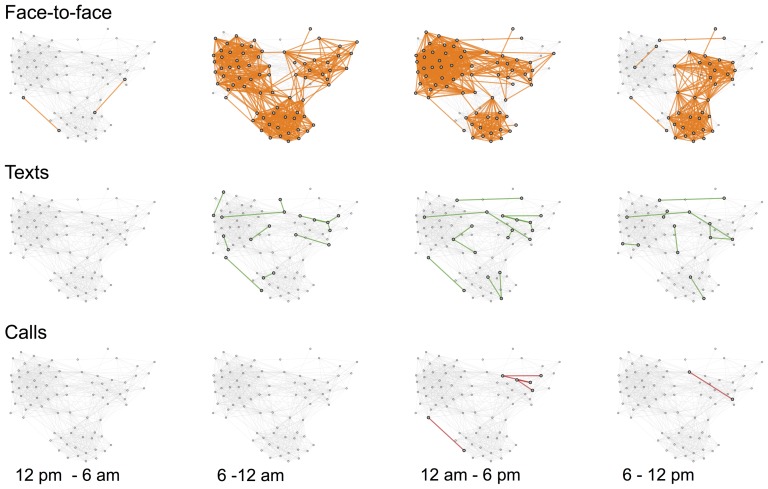
Daily activations in three networks. One day (Friday) in a network showing how different views are produced by observing different channels.

In Figure 11, we focus on a single day (Friday) and show activation of links between participants in three channels: voice calls, text messages, and face-to-face meetings. The three networks show very different views of the participants' social interactions.

### Online friendships

**Figure 12:**
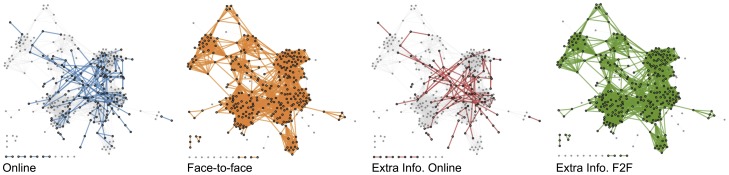
Face-to-face and online activity. The figure shows data from the 2013 deployment for one representative week. **Online**: Interactions (messages, wall posts, photos, etc.) between participants on Facebook. **Face-to-Face**: Only the most active edges, which account for 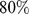 of all traffic, are shown for clarity. **Extra Info. F2F**: Extra information contained in the Bluetooth data shown as the difference in the set of edges. **Extra Info. Online**: Additional information contained in the Facebook data.

The past years have witnessed a shift in our interaction patterns, as we have adapted new forms of online communication. Facebook is to date the largest online social community with more than 1 billion users worldwide [167]. Collecting information about friendship ties and communication flows allows us to construct a comprehensive picture of the online persona. Combined with other recorded communication channels we have an unparalleled opportunity to piece together an almost complete picture of all major human communication channels. In the following section we consider Facebook data obtained from the 2013 deployment. In contrast to the first deployment, we also collected interaction data in this deployment. For a representative week (Oct. 14–Oct. 21, 2013), we collected 155 interactions (edges) between 157 nodes, yielding an average degree 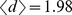, average clustering 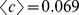, and average shortest path in the giant component (86 nodes) 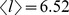. The network is shown in the left-most panel of Figure 12. By comparing with other channels we can begin to understand how well online social networks correspond to real life meetings. The corresponding face-to-face network (orange) is shown in Figure 12, where weak links, i.e. edges with fewer than 147 observations (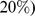 are discarded. Corresponding statistics are for the 307 nodes and 3 217 active edges: 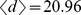, 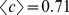, and 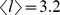. Irrespective of the large difference in edges, the online network still contains valuable information about social interactions that the face-to-face network misses—red edges in Figure 12.

**Figure 13:**
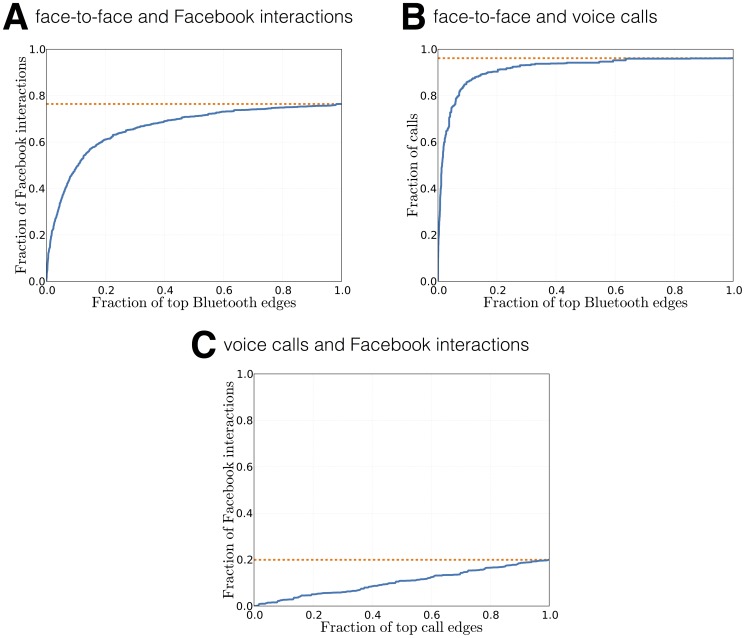
Network similarity. Defined as the fraction of ties from one communication channel that can be recovered by considering the top 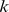 fraction of edges from a different channel. Orange dashed line indicates the maximum fraction of ties the network accounts for. The strongest 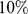 of face-to-face interactions account for 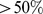 of online ties and 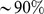 of call ties, while 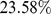 of Facebook ties and 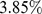 of call ties are not contained in the Bluetooth data. Between call and Facebook, the 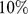 strongest call ties account for 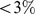 while in total 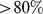 of Facebook ties are unaccounted. All values are calculated for interactions that took place in January 2014.

A simple method for quantifying the similarity between two networks is to consider the fraction of links we can recover from them. Sorting face-to-face edges according to activity (highest first) we consider the fraction of online ties the top 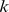 Bluetooth links correspond to. Figure 13A shows that 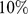 of the strongest Bluetooth ties account for more than 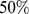 of the Facebook interactions. However, as noted before, the Bluetooth channel does not recover all online interactions—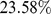 of Facebook ties are unaccounted for. Applying this measure between Bluetooth and voice calls (Figure 13B) shows a similar behavior, while there is low similarity between voice calls and Facebook ties (Figure 13C).

### Personality traits

While the data from mobile sensing and online social networks provide insights primarily into the structure of social ties, we are also interested in the demographics, psychological and health traits, and interests of the participants. Knowing these characteristics, we can start answering questions about the reasons for the observed network formation; why are ties created and what drives their dynamics? For example, homophily plays a vital role in how we establish, maintain, and destroy social ties [168].

**Figure 14:**
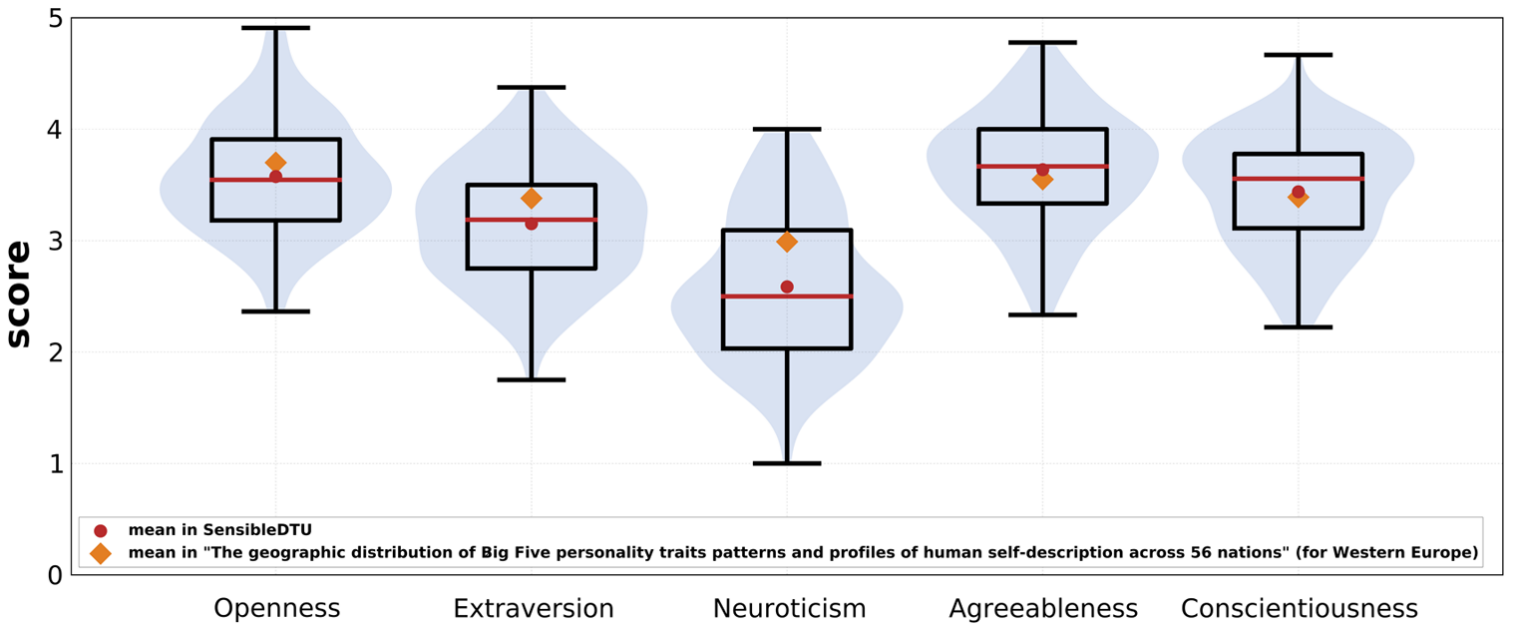
Personality traits. Violin plot of personality traits. Summary statistics are: **openness** 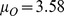, 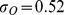; **extraversion** 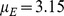, 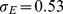; **neuroticism** 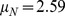 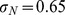; **agreeablenes** 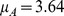 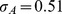; **conscientiousness** 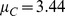 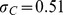. Mean values from our deployment (red circles) compared with mean values reported for Western Europe (mixed student and general population) [170] (orange diamonds).

Within the study, participants answered questions covering the aforementioned domains. These questions included the widely used *Big Five Inventory* [135] measuring five broad domains of human personality traits: openness, extraversion, neuroticism, agreeableness, and conscientiousness. The traits are scored on a 5-point Likert-type scale (low to high), and the average score of questions related to each personality domain are calculated. As Big Five has been collected for various populations, including a representative sample from Germany [169] and a representative sample covering students mixed with the general population from Western Europe [170], we report the results from the 2012 deployment in Figure 14, suggesting that our population is unbiased with respect to these important traits.

**Figure 15:**
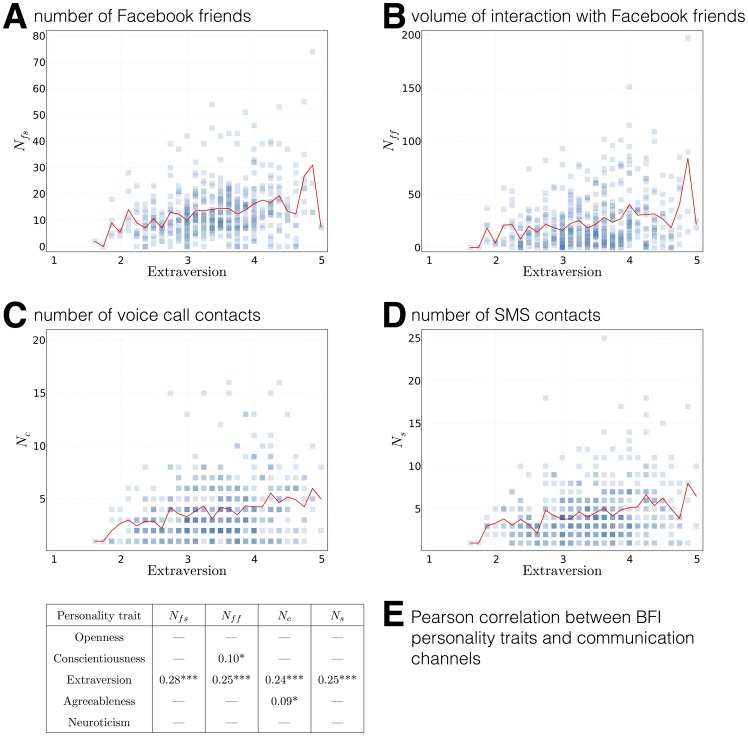
Correlation between personality traits and communication. Data from the 2013 deployment for N = 488 participants, showing communication only with other study participants. Extraversion, the only significant feature across all networks is plotted. The red line indicates mean value within personality trait. Random spikes are due to small number of participants with extreme values. **E**) Pearson correlation between Big Five Inventory personality traits and number of Facebook friends 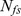, volume of interactions with these friends 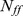, number of friends contacted via voice calls 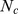 and via SMS 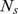. *: 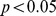, **: 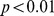, ***: 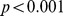.

Following the idea that personality is correlated with the structure of the social networks, we examine how the Big Five Inventory traits relate to the communication ego networks of the participants: number of Facebook friends, amount of communication with these friends, number of people ever contacted over voice calls or SMS. We only consider communication within the study, in the 2013 deployment for N = 488 participants for whom complete and longitudinal data was available. It is worth noting that participants answered the questions very early in the semester, and that we anecdotally know that a vast majority of the friendships observed between participants are ‘new’ in that they are between people who met when they started studying. Thus, we mainly observe the effect of personality on the network structure, not the other way around. The results are consistent with the literature, where Extraversion was shown to be correlated with number of Facebook friends [171]. Extending this result, Figure 15 depicts the correlation between Extraversion and number of Facebook friends (structural network) 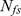 (Figure 15A), volume of interactions with these friends (functional network) 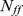 (Figure 15B), number of friends contacted via voice calls 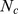 (Figure 15C), and number of friends contacted via SMS 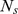 (Figure 15D). In Table 15E, we show the (Pearson) correlation between all five traits and the aforementioned communication channels, reporting only significant results. The values of correlation for Extroversion are consistent across the networks, and are close to those reported in [171], [172] (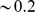). Following the result from Call & Text Communication Patterns Section, where we showed that the communication in SMS and call networks are similar in volume, however have limited overlap in terms of who participants contact, both those channels show similar correlation with Extraversion. Here, we only scratched the surface with regard to the relation between personality and behavioral data. The relation between different behavioral features, network structure, and personality has been studied in [173]–[176]. By showing the impact of Extraversion on the network formed with participants inside the study is consistent with values reported for general populations, we indicate that within the Copenhagen Networks Study, we capture a true social system, with different personalities positioned differently in the network.

## Perspectives

We expect that the amount of data collected about human beings will continue to increase. New and better services will be offered to users, more effective advertising will be implemented, and researchers will learn more about human nature. As the complexity and scale of studies on social systems studies grows, collection of high-resolution data for studying human behavior will become increasingly challenging on multiple levels, even when offset by the technical advancements. Technical preparations, administrative tasks, and tracking data quality are a substantial effort for an entire team, before even considering the scientific work of data analysis. It is thus an important challenge for the scientific community to create and embrace re-usable solutions, including best practices in privacy policies and deployment procedures, supporting technologies for data collection, handling, and analysis methods.

The results presented in this paper—while still preliminary considering the intended multi-year span of the project—clearly reveal that a single stream of data rarely supplies a comprehensive picture of human interactions, behavior, or mobility. At the same time, creating larger studies, in terms of number of participants, duration, channels observed, or resolution, is becoming expensive using the current approach. The interest of the participants depends on the value they get in return and the inconvenience the study imposes on their lives. The inconvenience may be measured by decreased battery life of their phones, annoyance of answering questionnaires, and giving up some privacy. The value, on the other hand, is classically created by offering material incentives, such as paying participants or, as in our case, providing smartphones and creating services for the participants. Providing material incentives for thousands or millions of people, as well as the related administrative effort of study management, may simply not be feasible.

In the not-so-distant future, many studies of human behavior will move towards accessing already existing personal data. Even today we can access mobility of large populations, by mining data from Twitter, Facebook, or Flickr. Or, with participants' authorizations, we can track their activity levels, using APIs of self-tracking services such as Fitbit or RunKeeper. Linking across multiple streams is still difficult today (the problem of data silos), but as users take more control over their personal data, scientific studies can become consumers rather than producers of the existing personal data.

This process will pose new challenges and amplify the existing ones, such as the replicability and reproducibility of the results or selection bias in the context of full end-user data control. Still, we expect that future studies will increasingly rely on the existing data, and it is important to understand how the incomplete view we get from such data influences our results. For this reason, we need research testbeds—such as the Copenhagen Networks Study—where we study ‘deep data’ in the sense of multi layered data streams, sampled with high temporal resolution. These deep data will allow us to unlock and understand the future streams of big data.
